# Atopic-eczema-associated fracture risk and oral corticosteroids: a population-based cohort study

**DOI:** 10.1016/j.jaip.2021.09.026

**Published:** 2021-09-24

**Authors:** Julian Matthewman, Kathryn E. Mansfield, Daniel Prieto-Alhambra, Amy R. Mulick, Liam Smeeth, Katherine E. Lowe, Richard J. Silverwood, Sinéad M. Langan

**Affiliations:** a Department of Non-Communicable Disease Epidemiology, London School of Hygiene and Tropical Medicine; b Health Data Research UK, London; c Centre for Statistics in Medicine, Nuffield Department of Orthopaedics, Rheumatology, and Musculoskeletal Sciences, University of Oxford, Oxford, OX3 7LD, UK; d Cleveland Clinic Lerner College of Medicine of Case Western Reserve School of Medicine, Cleveland, OH, USA; e Centre for Longitudinal Studies, University College London

**Keywords:** Atopic eczema, atopic dermatitis, fracture, osteoporotic fracture, oral corticosteroids

## Abstract

**Background:**

Evidence suggests adults with atopic eczema have increased fracture risk. However, it is unclear whether oral corticosteroids explain the association.

**Objective:**

To assess to what extent oral corticosteroids mediate the relationship between atopic eczema and fractures.

**Methods:**

We conducted a cohort study using English primary care (Clinical Practice Research Datalink) and hospital admissions (Hospital Episode Statistics) records (1998-2016) including adults (18+) with atopic eczema matched (age, sex, and general practice) with up to five adults without atopic eczema.

We used Cox regression to estimate hazard ratios (HRs) for specific major osteoporotic fractures (hip, spine, pelvis, wrist) and for any-site fracture comparing individuals with atopic eczema to those without, adjusting for six different definitions of time-updated oral corticosteroid use (ever any prescription, ever high dose, and recent, cumulative, current or peak dose).

**Results:**

We identified 526,808 individuals with atopic eczema and 2,569,030 without. We saw evidence of an association between atopic eczema and major osteoporotic fractures (e.g., spine HR 1.15 99%CI 1.08–1.22; hip HR 1.11 99%CI 1.08–1.15) that remained after additionally adjusting for oral corticosteroids (e.g., cumulative corticosteroid dose: spine HR 1.09 99%CI 1.03–1.16; hip HR 1.09 99%CI 1.06–1.12). Fracture rates were higher in people with severe atopic eczema compared to people without even after adjusting for oral corticosteroids (e.g., spine HR [99%CI]: confounder adjusted 2.31 [1.91–2.81]; additionally adjusted for cumulative dose 1.71 [1.40–2.09]).

**Conclusion:**

Our findings suggest that little of the association between atopic eczema and major osteoporotic fractures is explained by oral corticosteroid use.

## Introduction

Atopic eczema, also referred to as atopic dermatitis, is common (affecting up to 10% of adults),^1^ and characterised by rash and itching. Evidence indicates people with atopic eczema have increased fracture risk.^2–5^ Our previous study showed that people with atopic eczema have 10% higher fracture risk than those without, and risk increases substantially in people with severe atopic eczema (e.g., spinal fracture risk in people with severe atopic eczema was more than twice that in people without).^6^ Understanding the mechanisms behind the association between atopic eczema and fracture is important given the high morbidity and mortality associated with fractures.^7^ It is possible that oral corticosteroid use may explain fracture risk in people with atopic eczema. Although current guidelines for the treatment of atopic eczema generally discourage oral corticosteroid use,^8–10^ there is evidence of their frequent use in practice.^11–14^ Thus, some of the effect of atopic eczema on fracture risk could be mediated through oral corticosteroids, especially in those with more severe atopic eczema, who may be treated more frequently with oral corticosteroids. Asthma, a common comorbidity in people with atopic eczema, is also commonly treated with oral steroids,^15,16^ and consequently may confound the relationship between atopic eczema and fractures.

In our previous study of atopic eczema and fracture,^6^ we adjusted for both asthma and oral corticosteroid use (defined as never or ever having received a prescription for a high-dose oral corticosteroid, i.e., >=20mg prednisolone equivalent dose [PED]/day). However, oral corticosteroids are often prescribed dynamically in relapsing and remitting diseases such as atopic eczema and asthma, with changing doses and prescription lengths,^17^ so it is possible we did not adequately capture the mediating effect of oral corticosteroid use in our previous study. Further, recent evidence highlights the importance of incorporating more detailed definitions of exposure to oral corticosteroids when assessing fracture risks.^17^ Understanding the role of oral corticosteroid use is clinically relevant to clarify whether atopic eczema, in the absence of oral corticosteroid use, should be considered a risk factor in bone density screening guidelines.

Therefore, we aimed to explore the role of oral corticosteroid use in the relationship between atopic eczema and major osteoporotic fractures, including its role as a mediator using different definitions of oral corticosteroid use.

## Methods

### Study design and setting

We conducted a cohort study using primary care electronic health record data from the Clinical Practice Research Datalink (CPRD) and linked hospital admissions data from Hospital Episode Statistics (HES).

### Data sources

CPRD includes over 11 million people from 674 practices in the United Kingdom.^18^ The HES database contains information for all NHS-funded hospital admissions in England.^19^

### Study population

**Figure 1:**
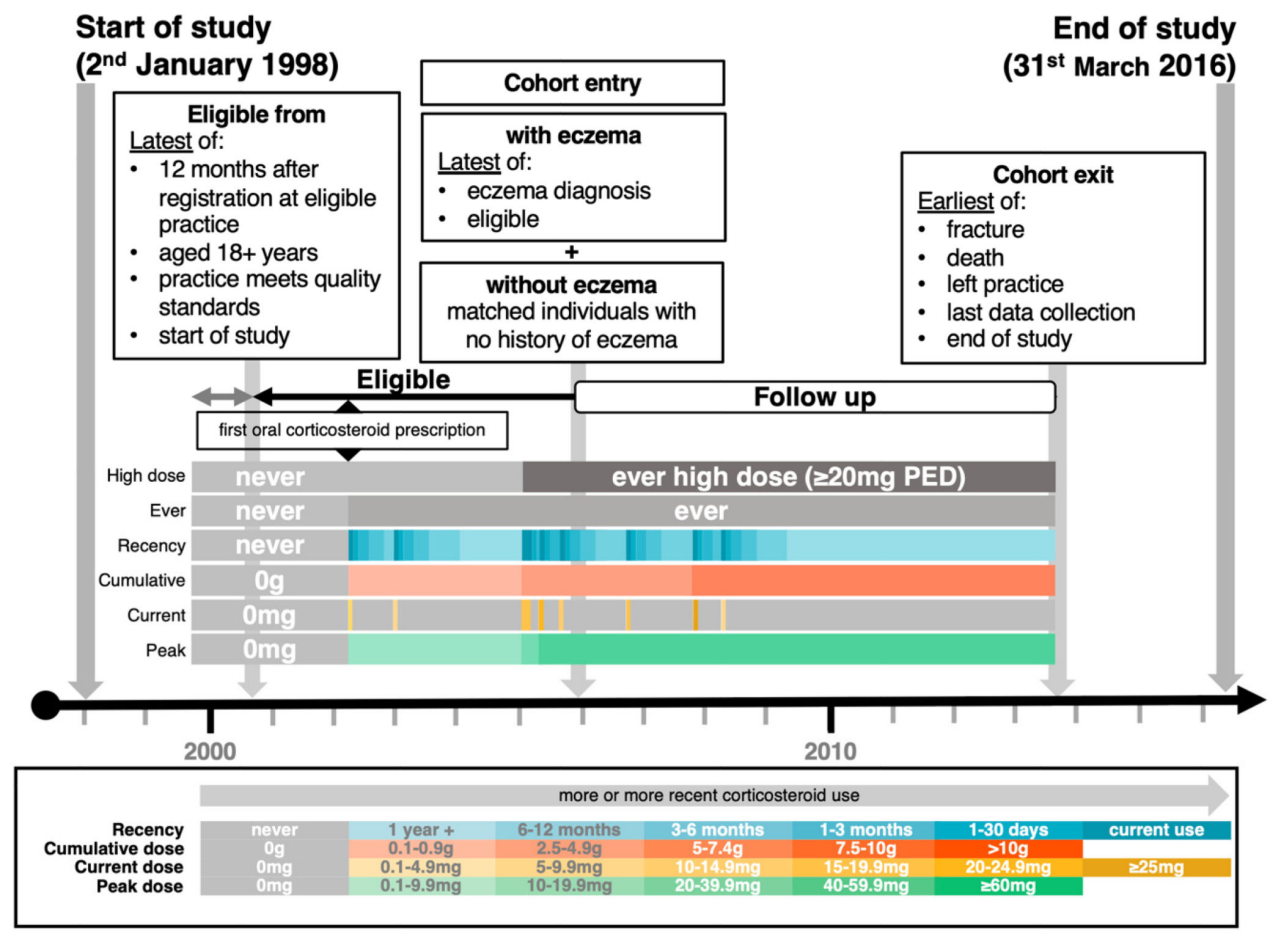
Illustration of the study cohort including an example of how corticosteroid use was captured over time, with lighter gradients representing less or less recent, and darker gradients representing more or more recent corticosteroid use.

Our study population included adults (18+ years) with at least one year of registration with a CPRD practice between January 2^nd^ 1998 and March 31^st^ 2016, who were eligible for HES linkage (England only). Individuals entered the atopic eczema cohort on the latest of the following: date atopic eczema diagnosis algorithm met, 18^th^ birthday, study start date (January 2^nd^ 1998), date their practice met CPRD quality-control standards, or practice registration date plus one year (to allow for the accurate capture of comorbidities and lifestyle factors). We included individuals with both prevalent and incident atopic eczema (dynamic cohort approach) (Figure 1).^20^

We matched individuals with atopic eczema (without replacement) with up to five randomly selected individuals without atopic eczema on age, sex, general practice, and date of cohort entry. Matched individuals without atopic eczema entered the cohort on the same date as the individual with atopic eczema they were matched to. People without atopic eczema with a subsequent morbidity code for atopic eczema contributed follow up time to the cohort without atopic eczema until their first record of an atopic eczema diagnosis. Participants were followed until the earliest of: fracture diagnosis (specific fracture of interest or any fracture site depending on the outcome analysed), death, departure from their practice, or practice no longer contributing to CPRD.

### Exposures, outcomes, and covariates

We defined atopic eczema (exposure) and atopic eczema severity (secondary exposure), fractures (outcome) and covariates using primary-care (Read codes) and secondary-care (International Classification of Diseases 10^th^ revision [ICD-10]) morbidity coding, and primary care prescriptions.^21^ Code lists for all study variables are available for download (https://doi.org/10.17037/DATA.00001156) and variable definitions were described in detail for our previous study.^6^

#### Atopic eczema

We identified people with atopic eczema, based on a validated algorithm, if they had at least one diagnostic code for atopic eczema and at least two records for atopic eczema treatment on separate days (i.e., topical corticosteroids, calcineurin inhibitors, cyclosporine, azathioprine, mycophenolate or methotrexate, or phototherapy).^21,22^

Participants with atopic eczema were assumed to have mild disease by default. They were identified as having moderate atopic eczema on the date they were prescribed either potent topical corticosteroids or topical calcineurin inhibitors, and severe atopic eczema when they were referred to a dermatologist, prescribed a systemic drug for the treatment of atopic eczema (i.e., azathioprine, cyclosporine, methotrexate, or mycophenolate mofetil, but not including oral corticosteroids), or had a record for phototherapy.^23,24^

#### Fracture

Our outcomes were specific major osteoporotic fractures: spine, hip (proximal femur), wrist and pelvis. Our “any fracture” outcome included any fracture site, but specifically excluded surgical, allograft, autograft, neoplasm-related and stress fractures, as these were considered to be unlikely to be related to atopic eczema.^6^

Participants were followed until they first experienced a fracture at the site under analysis. We excluded participants if they had a record for a previous fracture at the same site at any time-point before the start of follow up (e.g., in analyses of hip fractures, individuals were excluded if they had a hip fracture before cohort entry), as previous fractures greatly increase the risk of subsequent fracture at the same site.^25^

#### Covariates

We used quintiles of Index of Multiple Deprivation (IMD) to assess deprivation.^26^ We used individual-level IMD data and supplemented with practice-level IMD data if individual-level data were unavailable. We identified asthma (presence/absence) using primary care morbidity coding updating asthma status on the first date of a relevant diagnostic code. We defined body mass index (BMI) and smoking status (never/ever) using primary care records close to cohort entry, as described in detail for our previous study.^6,27^ We identified participants as harmful alcohol users from their first record for a morbidity code suggesting harmful alcohol use, or a prescription for drugs used to maintain abstinence. Justifications for the inclusion of covariates, and the basis of their categorisation are provided in the online repository (Text E1).

We identified primary care prescriptions for oral corticosteroids with glucocorticoid activity (prednisolone, betamethasone, deflazacort, dexamethasone, hydrocortisone, methylprednisolone, prednisone, triamcinolone, and cortisone) and calculated the PED in milligrams(mg)/day. In addition to the definition of high-dose oral corticosteroid use we used in our previous study (i.e., >=20mg PED/day),^6^ we identified five different time-updated measures of oral corticosteroid use: ever-prescribed an oral corticosteroid, recent dose, cumulative dose, current prescription and peak dose (Table 1).^17^

#### Modelling strategy

We used a directed acyclic graph (DAG) to visualise our *a priori* assumptions about the potential mechanisms explaining any link between atopic eczema and fractures, and to guide selection of confounders and mediators for use in adjusted regression models (eFigure 1). We drew paths using prior knowledge and existing literature.^1,28–34^

### Statistical analyses

#### Main analyses

We used Cox proportional hazards regression, stratifying on matched set, to estimate hazard ratios for the effect of atopic eczema on fractures. Our analyses implicitly adjusted for age, sex, practice, and date of cohort entry through matching and underlying timescale (age), and additionally adjusted (confounder adjusted model) for calendar time (1997-2001, 2002-2006, 2007-2011, 2012-2016), asthma and IMD quintiles. To estimate the effect of atopic eczema that was not mediated through oral corticosteroid use, we additionally adjusted for six different definitions of corticosteroid use (1. ever vs never; 2. high-dose [>20mg PED/day] vs never; 3. recent prescription; 4. cumulative dose; 5. current dose; and 6. peak dose) in six separate models.^17^

### Sensitivity analyses

We undertook sensitivity analyses to examine whether a different cumulative dose definition or the addition of ethnicity as a covariate affected our results (Table E4, Text E2, Table E5).

### Secondary analyses

#### Atopic eczema severity

To explore the effect of atopic eczema severity on fracture risk we compared people with time-updated mild/moderate/severe atopic eczema to people with no atopic eczema.

#### Rate differences

We calculated rate differences from fracture incidence rates for different fractures in those with and without atopic eczema. The incidence rate of specific fractures in participants without atopic eczema was estimated as the incidence rate of those with atopic eczema multiplied by the inverse of the hazard ratio of the confounder adjusted model with cumulative dose (r*(1/HR)) (Table E3).

We used 99% confidence intervals throughout the study to minimise the risk of type I error. We used Stata version 15 (StataCorp, College Station, Texas) for initial data management, and R version 3.5.2 for further data management and statistical analyses.^35,36,36^

The study was approved by CPRD’s Independent Scientific Advisory Committee (ISAC Protocol Number: 16_100RA).

## Results

**Figure 2:**
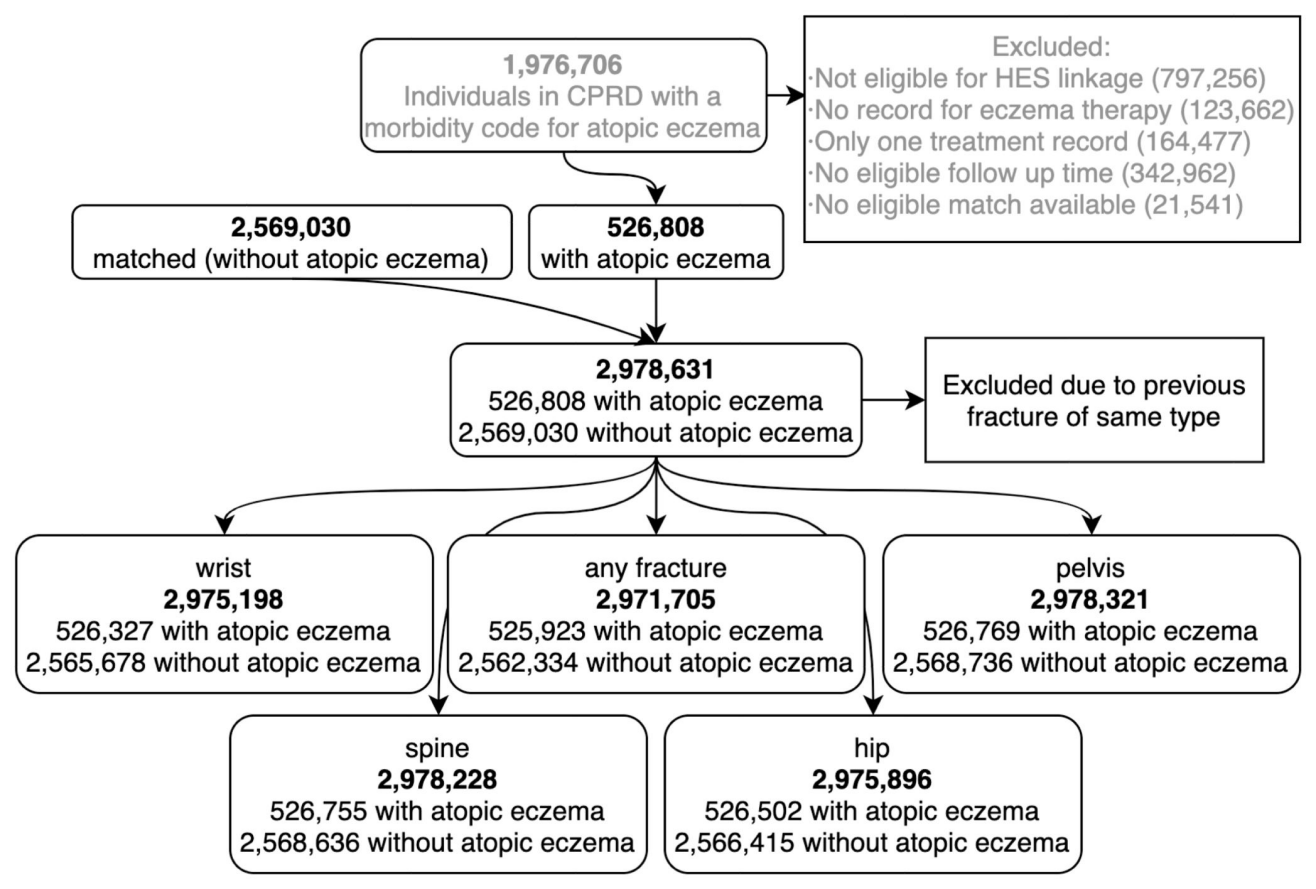
Study flow diagram. The dataset used for this study contained the 526,808 individuals with atopic eczema and matched 2,569,030 individuals without atopic eczema that remained after exclusions. Participants with a previous fracture at the same site were excluded. For the analysis of specific fractures, only those were excluded that had a previous fracture of the fracture type of interest.

We identified 525,923 individuals with atopic eczema and 2,562,334 matched participants without (Figure 2). Individuals with and without atopic eczema were broadly similar in terms of age, sex, BMI, smoking status and IMD (Table 2). Those with atopic eczema were more likely to have asthma (27.7% vs 15.0%) and at least one prescription for oral corticosteroids (27.8% vs 14.1%).

### Main analysis

**Figure 3:**
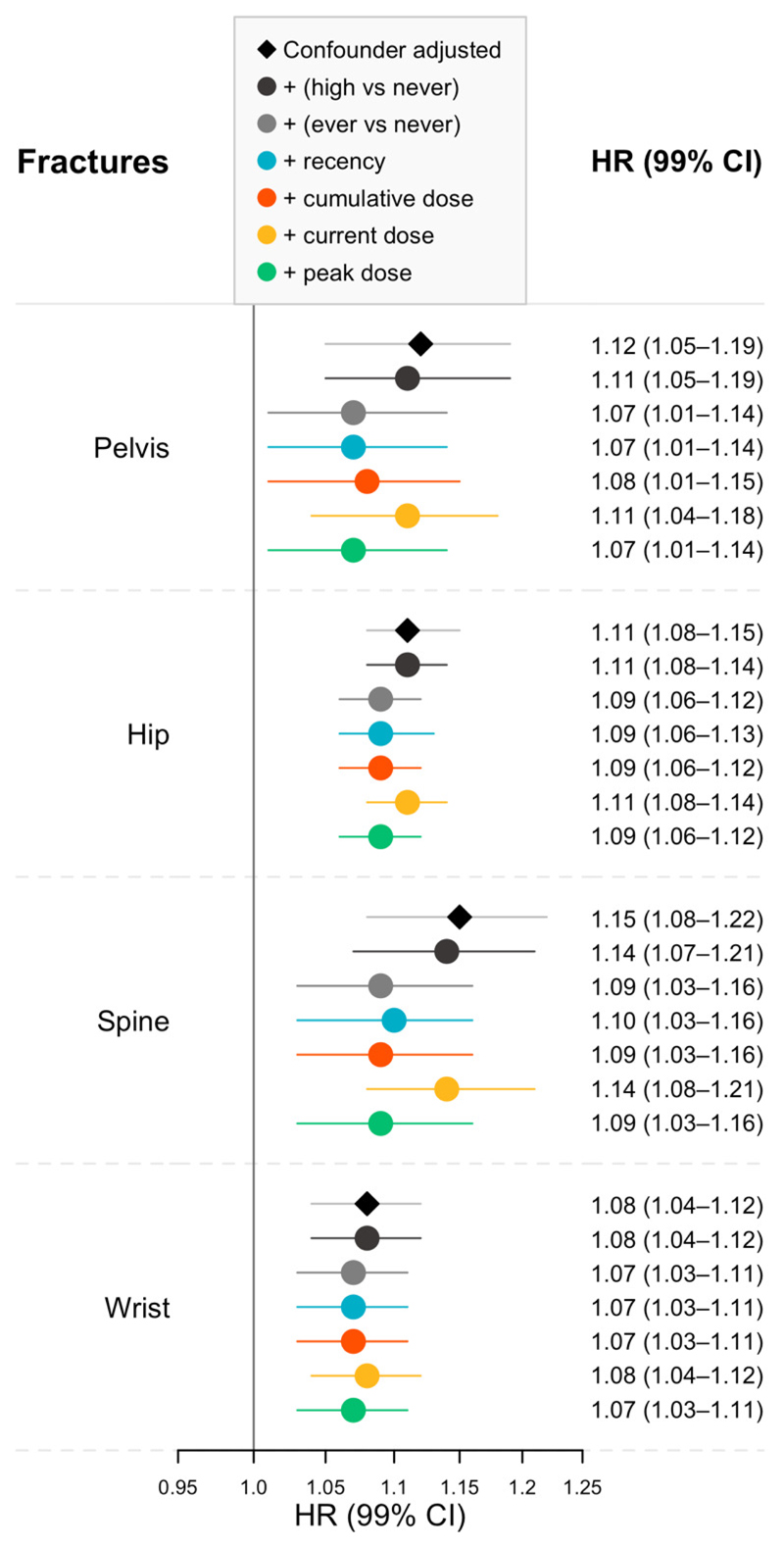
Hazard ratios (HR) with 99% confidence intervals (99% CI) for risk of fracture in people with atopic eczema compared to people without in confounder adjusted models additionally adjusted for different definitions of oral corticosteroid use. HRs estimated using Cox regression implicitly adjusted for age, sex, general practice, and date of cohort entry (due to matching and underlying timescale), and explicitly adjusted for calendar period, IMD and asthma (confounder adjusted). Number of fracture events recorded in those with atopic eczema: Spine 3,327; Hip 13,709; Pelvis: 3,151; Wrist 7,737.

In minimally-adjusted (implicitly adjusted for age, sex, general practice, and date of cohort entry) Cox models, those with atopic eczema compared to those without atopic eczema had higher risk of fracture (e.g., spine HR 1.19 99%CI 1.12-1.26; hip HR 1.13 99%CI 1.05-1.21). After additionally adjusting for calendar time, IMD and asthma, the effect of atopic eczema on fracture risk somewhat attenuated (e.g., spine HR 1.15 99%CI 1.08–1.22; hip HR 1.11 99%CI 1.08–1.15) (Table E1). After further adjusting for oral corticosteroid use there was still evidence of increased fracture risk in people with atopic eczema compared to those without (e.g., adjusted for never vs ever use: spine HR 1.09 99%CI 1.03–1.16; hip HR 1.09 99%CI 1.06–1.12) (Figure 3). Across all fracture sites, adjustment for high vs never and current dose corresponded to lower levels of attenuation while adjustment for the remaining definitions corresponded to greater levels of attenuation, though the extent of between-definition variability in attenuation differed by site and confidence intervals for different definitions overlapped.

#### Sensitivity analyses

Results from analyses using a different definition for cumulative corticosteroid dose were similar to those in the main analysis (Table E4). After additionally adjusting for ethnicity and restricting to individuals entering the cohort from 2006, effect estimates for the association between atopic eczema and pelvis, hip, spine, and wrist fractures were attenuated and confidence intervals crossed the null, in both confounder adjusted models and models additionally adjusting for cumulative oral corticosteroid dose (Text E2, Table E5). The restricted study population (with cohort entry dates from 2006) differed to the main study cohort, with individuals being on average younger (Table E6).

### Secondary analyses

#### Atopic eczema severity

**Figure 4:**
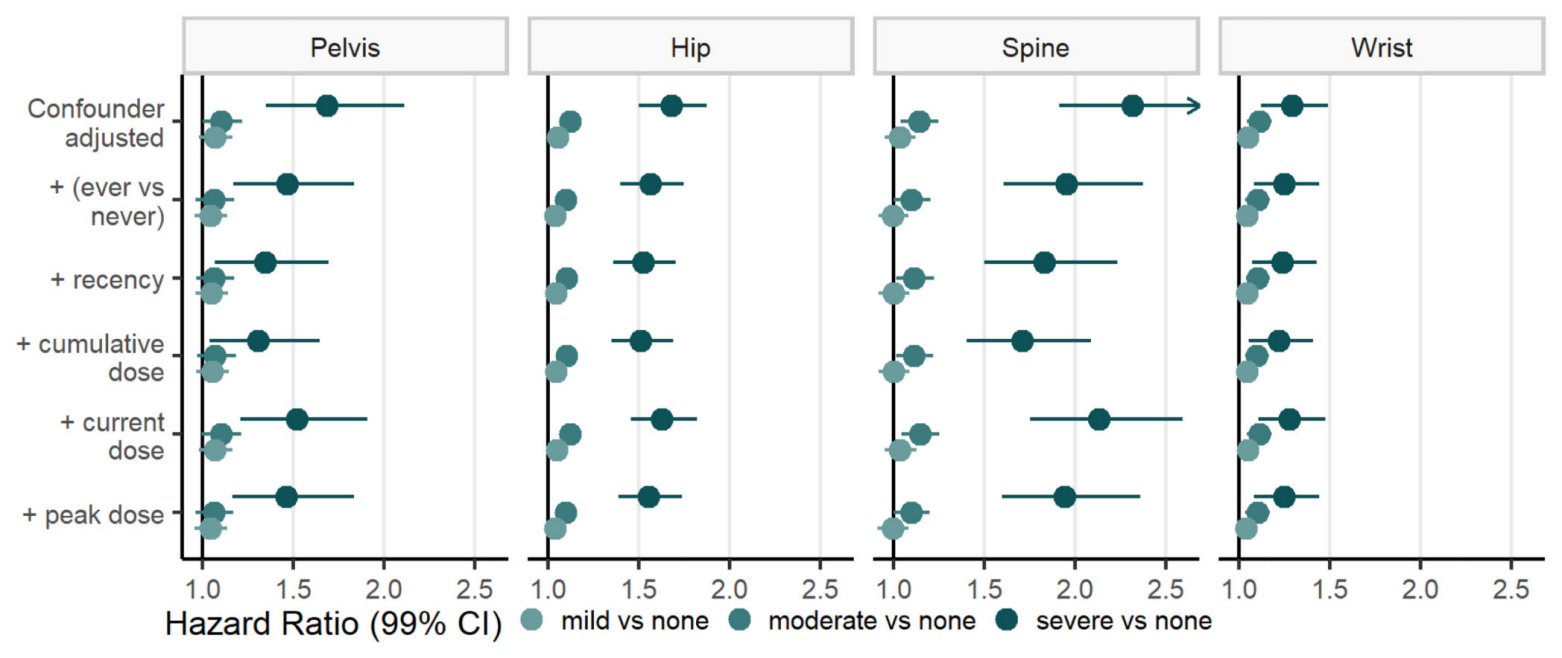
Hazard ratios with 99% confidence intervals (CI) comparing fracture risk for different fracture types in people with mild/moderate/severe atopic eczema to people without atopic eczema. Points with error bars are coloured by atopic eczema severity. Estimates from the confounder-adjusted models (implicitly adjusted for age, sex, general practice, and date of cohort entry, and explicitly adjusted for calendar period, IMD and asthma) and additionally adjusted for different definitions of oral corticosteroid use are shown. All estimates including estimates for any fracture site can be found in Appendix Table E2.

Fracture risk increased with increasing atopic eczema severity (e.g., confounder-adjusted HRs [99% CIs] for spinal fractures compared to no atopic eczema: mild 1.03 [0.95–1.12], moderate 1.14 [1.04–1.25], severe 2.31 [1.91–2.81]) (Figure 4, Table E2). Additionally adjusting for oral corticosteroid use somewhat attenuated effect estimates (e.g.: HRs [99% CIs] for spine fractures additionally adjusting for cumulative oral corticosteroid dose compared to no atopic eczema: mild 1.00 [0.92–1.08], moderate 1.11 [1.01–1.22], severe 1.71 [1.40–2.09]). Regardless of the definition used for oral corticosteroid use we saw a link between increasing severity of atopic eczema and increasing fracture risk.

#### Rate differences

After adjusting for confounders and oral corticosteroid use, we estimated that among those with atopic eczema between 0.07 (wrist) and 0.35 (hip) site-specific fractures per 1,000 person years were attributable to atopic eczema (Table E3).

## Discussion

We found evidence of an association between atopic eczema and major osteoporotic fractures that persisted after adjusting for oral corticosteroids regardless of how oral corticosteroid use was defined. The link between atopic eczema and fractures was greater in more severe atopic eczema and varied by fracture site, with spinal fractures being more than twice as common in those with severe atopic eczema compared to people without atopic eczema. Evidence of an association between atopic eczema and fracture risk remained after adjusting for oral corticosteroid use, indicating that only some of the association between atopic eczema and fracture risk could be explained by oral corticosteroid use. After adjusting for oral corticosteroids, the attenuation of the association between atopic eczema and fractures was greater in people with severe atopic eczema (i.e., when additionally adjusting for oral corticosteroids the effect estimate decreased more in people with severe atopic eczema than for those with moderate or mild atopic eczema), potentially due to more frequent use of oral corticosteroids in severe atopic eczema.

### Results in context

Our results offer insight into the role of oral corticosteroids in the association between atopic eczema and fracture. While a number of other studies describe an association between atopic eczema and worse bone health, insight into the impact of oral corticosteroids on the relationship, until now, has been limited, as described in a recent systematic review.^5^ Of the studies included in the review, only our previous study^6^ and one other population-based cohort study from Taiwan^2^ adjusted analyses for oral corticosteroid use, both using definitions that classified steroid use into categories of never or ever.

Results from a recent Danish study suggest that the use of high cumulative doses of potent topical corticosteroids could be associated with increased risk of major osteoporotic fractures, albeit with small effect sizes (3% relative risk increase per doubling of cumulative topical corticosteroid dose).^37^ The Danish study’s observed role of topical steroids in the association between atopic eczema and fractures could be explained by confounding by indication (i.e., those receiving the highest cumulative doses of topical steroids are also those with the most severe disease) or residual confounding through oral corticosteroids, BMI or other covariates that were not accounted for in the study. However, topical corticosteroids may still explain some of the association between atopic eczema and fracture risk that we found. Our definition of atopic eczema severity included prescriptions for topical corticosteroids, and may therefore, to some extent, have captured the effect of topical corticosteroids on the association between atopic eczema and fracture.

### Strengths and limitations

Our study uses a large population-based cohort from a data source that contains information on key variables. Using detailed definitions of oral corticosteroid use allowed us to address to what degree oral corticosteroid use might explain the previously observed relationship between atopic eczema and fracture risk.^5^

The results of our study are likely to be generalisable to the general population of England, as CPRD covers a population that is broadly representative of the English general population in terms of age, sex and ethnicity.^18^

The association we saw between atopic eczema and fracture, could be explained by mediation through other potential observed (i.e., harmful alcohol use, smoking, BMI) and unobserved mediators (Figure 2), potentially including physical activity, osteoporosis, sleep impairment, and fatigue or day-time sleepiness (due to night-time itching or the use of sedating antihistamines to manage the sleep problems associated with the itch of atopic eczema).^38^ Our study did not directly account for topical corticosteroid use. Accurate capture of topical steroid use using health record data is complex as it depends on prescribed dose, treatment adherence and skin integrity. However, we were able to partially account for the effect of topical corticosteroids in our atopic eczema severity analyses, as our moderate atopic eczema definition included potent topical corticosteroid prescriptions.

Atopic eczema is a relapsing and remitting condition; therefore, our severity definition may not have adequately captured changing disease severity over time (as our definition did not allow individuals to return to a lower severity status). In not allowing individuals with more severe atopic eczema to be return to a lower severity status, we may have wrongly classified individuals with milder atopic eczema as having more severe disease. However, this would only bias our results to the null, meaning that the findings of our severity analyses are likely to be underestimates of the true effect of atopic eczema severity on fracture risk.

We used routinely collected health records to define atopic eczema severity based on prescribed drugs and therapies, rather than a standardised clinical severity score (as these definitions are not available in routine data).^39^ Our severity definition has been used in previous electronic health record studies and demonstrates a similar distribution of mild/moderate/severe atopic eczema to those seen using clinical severity definitions. ^6,40,41^ Therefore, we believe our severity definition is appropriate in this context but encourage efforts to standardise severity definitions for electronic health records research.

However, there is some potential for misclassification of atopic eczema severity in our severity definition, possibly biasing effect estimates. Individuals can only step up in severity, but not step down (i.e., once an individual is classified as having moderate or severe atopic eczema, they cannot be reclassified as having a milder form of atopic eczema). Therefore, individuals with remitted disease may be misclassified as having more severe disease. Conversely, individuals with more severe atopic eczema may be misclassified as having milder disease if they do not regularly consult their doctor for help with their condition.

We identified time-updated cumulative oral corticosteroid dose at the beginning of each prescription, adding the dose of the entirety of the respective prescription duration on the date of prescription (so cumulative dose status changed at the beginning of the prescription that initiated the increase in cumulative dose). It is likely that time-updating cumulative dose status more frequently (e.g., daily) would have little impact on our estimates as most prescription lengths are not long enough to lead to multiple changes in categories (median prescription duration: 28 days). However, results were similar in sensitivity analyses using the alternative approach of updating cumulative dose at the end of each prescription (Table E4).

There is potential for wrongly identifying individuals as taking corticosteroid drugs due to them not adhering to prescribed corticosteroid treatment, and we were unable to assess adherence. Most participants receiving oral corticosteroid prescriptions received more than one prescription (60.8%), implying that previous prescriptions were used. There remains some potential for misclassification in those with the most infrequent prescriptions, however we attempted to mitigate this by using detailed time-updated definitions of oral corticosteroid use (recency, cumulative, current and peak dose), which limits the potential periods of misclassification to the length of the prescription.

Participants with atopic eczema were identified based on an algorithm, with a positive predictive value of 82%.^6,22^ It is possible that a small number of individuals without atopic eczema may have been wrongly identified as having atopic eczema. However, it is unlikely any misclassification of atopic eczema status is related to fracture recording, so our estimates will only be biased towards the null. Further, a sensitivity analysis in our previous study using a broader atopic eczema definition showed similar results.^6^ Fractures are seldomly missed in primary or secondary care. However, spinal fractures, can go undiagnosed.^42,43^ Spinal fractures are more likely to be detected for participants with more frequent general practice (GP) consultations, which is likely to be the case for those with atopic eczema, and especially for those with severe atopic eczema. Some of the effect of atopic eczema on spinal fractures might therefore be explained by more frequent GP attendance. However, a sensitivity analysis in our previous study, restricting to participants that had attended their GP practice in the year before cohort entry, found only minimal differences in results.^6^

We did not explore the relationship between atopic eczema and osteoporosis as osteoporosis diagnoses and results from bone mineral density measurements are not robustly and systematically captured in routine health data, with higher risk individuals more likely to have a record for either. There is potential for residual confounding in this study. Data from CPRD do not provide robust information on vitamin D level, food allergy or intolerance, malnourishment or eating disorders.^6^

### Clinical interpretation

Our results indicate that the increased fracture risk in people with atopic eczema cannot be explained by oral corticosteroid use alone. Explanations for the link we saw between atopic eczema and fracture include chronic inflammation associated with atopic eczema, changed diet linked to food intolerances, or avoidance of physical activity as sweating can exacerbate atopic eczema symptoms, leading to osteoporosis and in turn to fractures.^44^ Other possible explanations for increased fracture risk are increased rates of harmful alcohol use or the use of sedating antihistamines leading to falls. The fracture outcomes used in this study are considered to be commonly associated with osteoporosis.^45^ Major osteoporotic fractures are associated with high morbidity and mortality, leading to, amongst other things, immobility, restriction of activities of daily living and thromboembolic disease. If atopic eczema increases the risk of these fractures, then considering including atopic eczema in guidelines for fracture prevention and encouraging the appropriate preventive care could substantially reduce fracture-related morbidity and mortality in people with atopic eczema. Given that atopic eczema is common, preventing associated fractures would represent an important public health intervention.

Importantly, our results do not suggest that oral corticosteroids do not contribute to at least some of the association between atopic eczema and fracture, or that oral corticosteroids for atopic eczema management is unproblematic. In line with current atopic eczema management guidelines, which reserve the use of oral corticosteroids for exceptional circumstances, clinicians should continue to avoid oral corticosteroids for atopic eczema.^10^

In sensitivity analyses additionally adjusting for ethnicity and restricting to those entering the study population from 2006 onwards (when ethnicity data was more likely to be complete^46^), our effect estimates were attenuated. This attenuation of effect after adjusting for ethnicity may be explained by the younger population in the restricted sample (Text E2, Table E6).

### Policy implications and future research

Current guidance recommends fracture-risk screening in people taking oral corticosteroids but does not specifically reference atopic eczema.^47–49^ Our results indicate that atopic eczema, especially severe atopic eczema, should be considered for inclusion in fracture-risk screening guidelines. Further research should explore why there is a link between atopic eczema and fracture, including the role of topical corticosteroids.

## Conclusion

In summary, we found that the association between atopic eczema and major osteoporotic fractures was not explained by oral corticosteroid use. Consideration should be given to adding atopic eczema to fracture risk screening guidance.

## Supplementary Material

Online repository

## Figures and Tables

**Table 1:** Different oral corticosteroid use definitions.

Definition	Description	Categories
Original high-dose oral corticosteroid definition^6^	Ever or never having received a prescription for ≥20mg/day PED. Updated on the date of the first recorded high-dose oral corticosteroid prescription.	never, ever
Ever versus never	Ever or never having received any prescription for oral corticosteroids. Updated on the date of the first recorded prescription.	never, ever
Recency	Has a current active prescription (i.e., between start and end of prescription date), or time since the last prescription end date.	never, 1 year+ (>365 days) 6-12 months (181-365 days) 3-6 months (91-180 days) 1-3 months (31-90 days) 1-30 days current
Cumulative dose	Sum of all corticosteroid doses prescribed, based on prescription length and daily PED. Updated at the start of prescriptions, adding the entirety of the prescription dose to the cumulative dose at once.	0 g 0.1 to 0.9 g 1 to 2.4 g 2.5 to 4.9 g 5 to 7.4 g 7.5 to 9.9 g ≥ 10 g
Current dose	Prescribed daily PED, categorised at start of an oral corticosteroid prescription, and then set back to zero at the end of the prescription.	0 mg (i.e.: no current prescription) 0.1 to 4.9 mg 5 to 9.9 mg 10 to 14.9 mg 15 to 19.9 mg 20 to 24.9 mg ≥25 m g
Peak dose	Highest daily PED recorded. Category updated if a prescription with a higher dose than any previous prescription was recorded.	s0 mg 0.1 to 9.9 mg 10 to 19.9 mg 20 to 39.9 mg 40 to 59.9 mg ≥60 mg

Abbreviation: PED (prednisolone equivalent dose).

**Table 2:** Characteristics of participants (data are n [%] unless otherwise specified).

	Without atopic eczema N=2,569,030		With atopic eczema N= 526,808	
**Age (years)**				
18–39	1,217,722	(47.4%)	246,596	(46.8%)
40–49	351,927	(13.7%)	69,696	(13.2%)
50–59	329,007	(12.8%)	63,943	(12.1%)
60–69	303,790	(11.8%)	61,902	(11.8%)
70+	366,584	(14.3%)	84,671	(16.1%)
**Sex** female	1,489,261	(58.0%)	308,071	(58.5%)
**IMD** ^1^				
1 (least deprived)	611,904	(23.8%)	126,806	(24.1%)
2	589,313	(22.9%)	120,946	(23.0%)
3	508,469	19.8%)	103,646	(19.7%)
4	489,144	19.0%)	100,430	(19.1%)
5 (most deprived)	370,200	14.4%)	74,980	(14.2%)
**Total follow up**, p-yrs (%)	14,118,405	(100%)	3,102,202	(100%)
**Median follow up**, years (IQR)	4.41	(1.70-8.90)	5.02	(2.00-9.64)
**Asthma**, p-yrs (%)	1,872,813	12.5%)	780,567	(23.6%)
**Any oral steroids**, p-yrs (%)	1,585,726	10.6%)	723,365	(21.9%)
**High-dose oral steroids^2^**, p-yrs (%)	849,832	5.7%)	396,332	(12.0%)
**Cumulative dose**, p-yrs (%)				
0 g	12,535,009	(83.9%)	2,379,540	(71.9%)
0.1 to 0.9 g	1,108,362	7.4%)	503,272	(15.2%)
1 to 2.4 g	204,979	1.4%)	102,161	(3.1%)
2.5 to 4.9 g	110,409	0.7%)	50,918	(1.5%)
5 to 7.4 g	50,360	0.3%)	22,065	(0.7%)
7.5 to 10 g	29,600	0.2%)	12,731	(0.4%)
>10 g	79,687	0.5%)	31,515	(1.0%)
**Peak dose**, p-yrs (%)				
0m g	12,535,610	(83.9%)	2,379,748	(71.9%)
0.1 to 9.9 mg	368,976	2.5%)	150,459	(4.5%)
10 to 19.9 mg	159,699	1.1%)	68,647	(2.1%)
20 to 39.9 mg	747,918	5.0%)	349,456	(10.6%)
40 to 59.9 mg	294,671	2.0%)	148,464	(4.5%)
>60 m g	11,532	0.1%)	5,428	(0.2%)

Abbreviation: p-yrs=person-years Age, sex, and IMD assessed at the beginning of follow-up. Person-years throughout follow-up are displayed for time-updated variables.

1 Quintiles of the Index of multiple deprivation (IMD)

2 High dose oral corticosteroids is defined as ever having been prescribed a prednisolone equivalent dose of >20mg/day.

## Highlights

### What is already known about this topic?

Adults with atopic eczema, especially those with severe atopic eczema, are at increased risk of major osteoporotic fracture, however, it is unclear what drives this association.

### What does this article add to our knowledge?

The association between atopic eczema and major osteoporotic fractures cannot be explained by oral corticosteroid use.

### How does this study impact current management guidelines?

Atopic eczema, especially severe atopic eczema, should be considered for inclusion in fracture-risk screening guidelines.

## Abbreviations

HR: … Hazard ratio
CI: … Confidence interval
PED: … Prednisolone equivalent dose
CPRD: … Clinical Practice Research Datalink
HES: … Hospital Episode Statistics
NHS: … National Health Service
ICD: … International Classification of Diseases
IMD: … Index of Multiple Deprivation
BMI: … Body mass index
DAG: … Directed acyclic graph
GP: … general practice
UK: … United Kingdom

## References

[1] Silverberg JI, Hanifin JM (2013). Adult eczema prevalence and associations with asthma and other health and demographic factors: A US population–based study. Journal of Allergy and Clinical Immunology.

[2] Wu C-Y, Lu Y-Y, Lu C-C, Su Y-F, Tsai T-H, Wu C-H, Nguyen TV (2017). Osteoporosis in adult patients with atopic dermatitis: A nationwide population-based study. Plos One.

[3] Garg NK, Silverberg JI (2015). Eczema is associated with osteoporosis and fractures in adults: A US population-based study. Journal of Allergy and Clinical Immunology.

[4] Garg N, Silverberg JI (2015). Association Between Eczema and Increased Fracture and Bone or Joint Injury in Adults: A US Population-Based Study. JAMA Dermatology.

[5] Mukovozov IM, Morra DE, Giustini D, Tadrous M, Cheung AM, Drucker AM (2020). Atopic dermatitis and bone health: a systematic review. Journal of the European Academy of Dermatology and Venereology [Internet].

[6] Lowe KE, Mansfield KE, Delmestri A, Smeeth L, Roberts A, Abuabara K (2020). Atopic eczema and fracture risk in adults: A population-based cohort study. Journal of Allergy and Clinical Immunology.

[7] Shuid AN, Khaithir TMN, Mokhtar SA, Mohamed IN (2014). A systematic review of the outcomes of osteoporotic fracture patients after hospital discharge: morbidity, subsequent fractures, and mortality. Therapeutics and Clinical Risk Management.

[8] Wollenberg A, Barbarot S, Bieber T, Christen-Zaech S, Deleuran M, Fink-Wagner A (2018). Consensus-based European guidelines for treatment of atopic eczema (atopic dermatitis) in adults and children: part I. Journal of the European Academy of Dermatology and Venereology.

[9] Wollenberg A, Barbarot S, Bieber T, Christen-Zaech S, Deleuran M, Fink-Wagner A (2018). Consensus-based European guidelines for treatment of atopic eczema (atopic dermatitis) in adults and children: part II. Journal of the European Academy of Dermatology and Venereology.

[10] Drucker AM, Eyerich K, de Bruin-Weller MS, Thyssen JP, Spuls PI, Irvine AD (2018). Use of systemic corticosteroids for atopic dermatitis: International Eczema Council consensus statement. British Journal of Dermatology.

[11] Yu SH, Drucker AM, Lebwohl M, Silverberg JI (2018). A systematic review of the safety and efficacy of systemic corticosteroids in atopic dermatitis. Journal of the American Academy of Dermatology.

[12] Simon D, Bieber T (2014). Systemic therapy for atopic dermatitis. Allergy.

[13] Ring J, Alomar A, Bieber T, Deleuran M, Fink-Wagner A, Gelmetti C (2012). Guidelines for treatment of atopic eczema (atopic dermatitis) Part II: Guidelines for treatment of atopic eczema. Journal of the European Academy of Dermatology and Venereology.

[14] Alexander T, Maxim E, Cardwell LA, Chawla A, Feldman SR (2018). Prescriptions for atopic dermatitis: oral corticosteroids remain commonplace. Journal of Dermatological Treatment.

[15] Gaga M, Zervas E (2019). Oral steroids in asthma: a double-edged sword. European Respiratory Journal.

[16] Ramsahai JM, Wark PA (2018). Appropriate use of oral corticosteroids for severe asthma. Medical Journal of Australia.

[17] Robinson DE, van Staa TP, Dennison EM, Cooper C, Dixon WG (2018). The limitations of using simple definitions of glucocorticoid exposure to predict fracture risk: A cohort study. Bone.

[18] Herrett E, Gallagher AM, Bhaskaran K, Forbes H, Mathur R, van Staa T (2015). Data Resource Profile: Clinical Practice Research Datalink (CPRD). International Journal of Epidemiology.

[19] Hospital Episode Statistics (HES).

[20] Vandenbroucke J, Pearce N (2015). Point: Incident Exposures, Prevalent Exposures, and Causal Inference: Does Limiting Studies to Persons Who Are Followed From First Exposure Onward Damage Epidemiology?. American Journal of Epidemiology.

[21] Lowe KE, Mansfield KE, Delmestri A, Smeeth L, Roberts A, Abuabara K (2019). Code lists for “Atopic eczema and fracture risk in adults: a population-based cohort study”.

[22] Abuabara K, Magyari AM, Hoffstad O, Jabbar-Lopez ZK, Smeeth L, Williams HC (2017). Development and Validation of an Algorithm to Accurately Identify Atopic Eczema Patients in Primary Care Electronic Health Records from the UK. Journal of Investigative Dermatology.

[23] Silverwood RJ, Forbes HJ, Abuabara K, Ascott A, Schmidt M, Schmidt SAJ (2018). Severe and predominantly active atopic eczema in adulthood and long term risk of cardiovascular disease: population based cohort study. BMJ.

[24] NICE Clinical guidance (2004). Tacrolimus and pimecrolimus for atopic eczema.

[25] Gehlbach S, Saag KG, Adachi JD, Hooven FH, Flahive J, Boonen S (2012). Previous fractures at multiple sites increase the risk for subsequent fractures: The global longitudinal study of osteoporosis in women. Journal of Bone and Mineral Research.

[26] Jordan H (2004). The Index of Multiple Deprivation 2000 and accessibility effects on health. Journal of Epidemiology & Community Health.

[27] Bhaskaran K, Douglas I, Forbes H, dos-Santos-Silva I, Leon DA, Smeeth L (2014). Body-mass index and risk of 22 specific cancers: a population-based cohort study of 5·24 million UK adults. The Lancet.

[28] Compston J (2018). Glucocorticoid-induced osteoporosis: an update. Endocrine.

[29] Hammer-Helmich L, Linneberg A, Thomsen SF, Glümer C (2014). Association between parental socioeconomic position and prevalence of asthma, atopic eczema and hay fever in children. Scandinavian Journal of Public Health.

[30] Iwaniec UT, Turner RT (2016). Influence of body weight on bone mass, architecture and turnover. Journal of Endocrinology.

[31] Ward KD, Klesges RC (2001). A meta-analysis of the effects of cigarette smoking on bone mineral density. Calcified Tissue International.

[32] Kantor R, Kim A, Thyssen JP, Silverberg JI (2016). Association of atopic dermatitis with smoking: A systematic review and meta-analysis. Journal of the American Academy of Dermatology.

[33] Berg KM, Kunins HV, Jackson JL, Nahvi S, Chaudhry A, Harris KA (2008). Association Between Alcohol Consumption and Both Osteoporotic Fracture and Bone Density. The American Journal of Medicine.

[34] Al-Jefri K, Newbury-Birch D, Muirhead CR, Gilvarry E, Araújo-Soares V, Reynolds NJ (2017). High prevalence of alcohol use disorders in patients with inflammatory skin diseases. British Journal of Dermatology.

[35] R Core Team (2018). R: A language and environment for statistical computing [Internet].

[36] Therneau TM, Grambsch PM (2000). Modeling survival data: extending the Cox model.

[37] Egeberg A, Schwarz P, Harsløf T, Andersen YMF, Pottegård A, Hallas J (2021). Association of Potent and Very Potent Topical Corticosteroids and the Risk of Osteoporosis and Major Osteoporotic Fractures.

[38] Behrendt H, Ring J (1990). Histamine, antihistamines and atopic eczema. Clinical & Experimental Allergy.

[39] Chopra R, Vakharia PP, Sacotte R, Patel N, Immaneni S, White T (2017). Severity strata for Eczema Area and Severity Index (EASI), modified EASI, Scoring Atopic Dermatitis (SCORAD), objective SCORAD, Atopic Dermatitis Severity Index and body surface area in adolescents and adults with atopic dermatitis. Br J Dermatol.

[40] Mansfield KE, Schmidt SAJ, Darvalics B, Mulick A, Abuabara K, Wong AYS (2020). Association Between Atopic Eczema and Cancer in England and Denmark. JAMA Dermatol.

[41] Schonmann Y, Mansfield KE, Hayes JF, Abuabara K, Roberts A, Smeeth L (2020). Atopic Eczema in Adulthood and Risk of Depression and Anxiety: A Population-Based Cohort Study. The Journal of Allergy and Clinical Immunology: In Practice.

[42] Delmas PD, van de Langerijt L, Watts NB, Eastell R, Genant H, Grauer A (2004). Underdiagnosis of Vertebral Fractures Is a Worldwide Problem: The IMPACT Study. Journal of Bone and Mineral Research.

[43] Hatgis J, Granville M, Jacobson RE (2017). Delayed Recognition of Thoracic and Lumbar Vertebral Compression Fractures in Minor Accident Cases. Cureus.

[44] Mundy GR (2008). Osteoporosis and Inflammation. Nutrition Reviews.

[45] Warriner AH, Patkar NM, Curtis JR, Delzell E, Gary L, Kilgore M (2011). Which fractures are most attributable to osteoporosis?. Journal of Clinical Epidemiology.

[46] Mathur R, Bhaskaran K, Chaturvedi N, Leon DA, vanStaa T, Grundy E (2014). Completeness and usability of ethnicity data in UK-based primary care and hospital databases. Journal of Public Health.

[47] Hippisley-Cox J, Coupland C (2012). Derivation and validation of updated QFracture algorithm to predict risk of osteoporotic fracture in primary care in the United Kingdom: prospective open cohort study. BMJ.

[48] Kanis JA, Oden A, Johansson H, Borgström F, Ström O, McCloskey E (2009). FRAX® and its applications to clinical practice. Bone.

[49] NICE Clinical guidance (2017). Osteoporosis: assessing the risk of fragility fracture.

