# Supplementary material for: Atopic-eczema-associated fracture risk and oral corticosteroids: a population-based cohort study
Source: J Allergy Clin Immunol Pract. Author manuscript; Available in PMC 2022 Jan 11. (PMC7612204; doi:10.1016/j.jaip.2021.09.026)
Supplement: Online repository [file EMS136900-supplement-Online_repository.docx]

## Online repository (for Atopic-eczema-associated fracture risk and oral corticosteroids: a population-based cohort study)

### Additional Tables, Figures, and Text


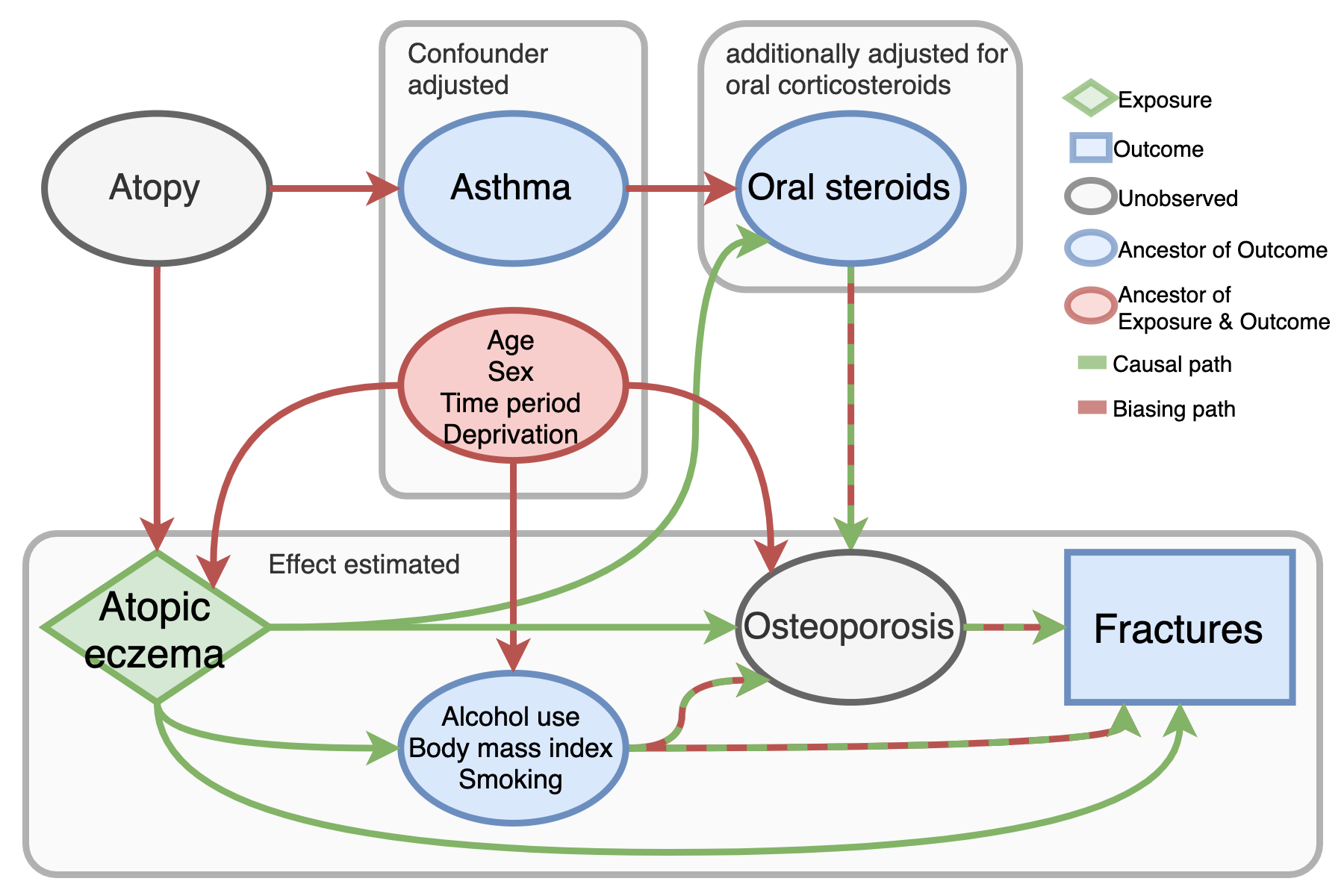


eFigure 1: Directed acyclic graph (DAG), showing the hypothesised relationships between exposure (eczema), outcome (fractures) and measured and unmeasured covariates. The variables that are included in the main study models are marked as is the effect estimated when adjusting for these variables.

Table E1: Hazard ratios (99% CIs) for the for risk of fracture comparing people with atopic eczema to people without atopic eczema in minimally adjusted models, confounder adjusted models and additionally adjusted for different steroid definitions.

| **fracture** | **person years** | **fractures (in those with atopic eczema)** | **fracture rate per 1000 p-yrs** | **Minimally adjusted** | **Confounder adjusted^1^** | **+ simple steroid definition (ever vs never)** | **+ recency of oral steroid prescription** | **+ cumulative dose of oral steroids** | **+ current dose of oral steroids** | **+ peak dose of oral steroids** |
| --- | --- | --- | --- | --- | --- | --- | --- | --- | --- | --- |
| pelvis | 3,309,099 | 3,151 | 0.95 | 1.14 (1.07–1.21) | 1.12 (1.05–1.19) | 1.07 (1.01–1.14) | 1.07 (1.01–1.14) | 1.08 (1.01–1.15) | 1.11 (1.04–1.18) | 1.07 (1.01–1.14) |
| hip | 3,307,483 | 13,709 | 4.14 | 1.13 (1.10–1.16) | 1.11 (1.08–1.15) | 1.09 (1.06–1.12) | 1.09 (1.06–1.13) | 1.09 (1.06–1.12) | 1.11 (1.08–1.14) | 1.09 (1.06–1.12) |
| spine | 3,308,947 | 3,327 | 1.01 | 1.19 (1.12–1.26) | 1.15 (1.08–1.22) | 1.09 (1.03–1.16) | 1.10 (1.03–1.16) | 1.09 (1.03–1.16) | 1.14 (1.08–1.21) | 1.09 (1.03–1.16) |
| wrist | 3,300,886 | 7,737 | 2.34 | 1.11 (1.07–1.15) | 1.08 (1.04–1.12) | 1.07 (1.03–1.11) | 1.07 (1.03–1.11) | 1.07 (1.03–1.11) | 1.08 (1.04–1.12) | 1.07 (1.03–1.11) |
| any site | 3,102,202 | 44,959 | 14.49 | 1.14 (1.12–1.16) | 1.11 (1.09–1.13) | 1.09 (1.07–1.11) | 1.09 (1.08–1.11) | 1.09 (1.08–1.11) | 1.11 (1.09–1.13) | 1.09 (1.07–1.11) |

Total person years and number of fractures and crude rates per 1000 person years are shown.
^1^implicitly adjusted for age, sex, general practice, and date of cohort entry, and additionally adjusted for calendar period (1997-2001, 2002-2006, 2007-2011, 2012-2016), quintiles of index of multiple deprivation and asthma.

Table E2: Hazard ratios (99% CIs) for the for risk of fracture comparing people with mild/moderate/severe atopic eczema to people without atopic eczema in minimally adjusted models, confounder adjusted models and additionally adjusted for different steroid definitions.

| **term** | **person years** | **fractures (in those with atopic eczema)** | **fracture rate per 1000 p-yrs** | **Minimally adjusted** | **Confounder adjusted^1^** | **+ simple steroid definition (ever vs never)** | **+ recency of oral steroid prescription** | **+ cumulative dose of oral steroids** | **+ current dose of oral steroids** | **+ peak dose of oral steroids** |
| --- | --- | --- | --- | --- | --- | --- | --- | --- | --- | --- |
| **pelvis** |  |  |  |  |  |  |  |  |  |  |
| mild | 2,000,441 | 1,636 | 0.82 | 1.08 (0.99–1.18) | 1.07 (0.98–1.16) | 1.04 (0.95–1.13) | 1.05 (0.96–1.14) | 1.05 (0.96–1.15) | 1.07 (0.98–1.16) | 1.04 (0.95–1.13) |
| moderate | 1,140,433 | 1,244 | 1.09 | 1.12 (1.02–1.24) | 1.10 (1.00–1.22) | 1.06 (0.96–1.17) | 1.06 (0.96–1.18) | 1.07 (0.97–1.18) | 1.10 (1.00–1.21) | 1.06 (0.96–1.17) |
| severe | 168,225 | 271 | 1.61 | 1.75 (1.40–2.19) | 1.69 (1.35–2.11) | 1.46 (1.17–1.83) | 1.34 (1.07–1.69) | 1.31 (1.04–1.64) | 1.52 (1.21–1.91) | 1.46 (1.16–1.83) |
| **hip** |  |  |  |  |  |  |  |  |  |  |
| mild | 1,999,680 | 6,825 | 3.41 | 1.06 (1.02–1.11) | 1.05 (1.01–1.10) | 1.04 (1.00–1.08) | 1.04 (1.00–1.08) | 1.04 (1.00–1.08) | 1.05 (1.01–1.09) | 1.04 (1.00–1.08) |
| moderate | 1,139,706 | 5,771 | 5.06 | 1.14 (1.09–1.19) | 1.12 (1.07–1.17) | 1.10 (1.05–1.15) | 1.10 (1.05–1.15) | 1.10 (1.05–1.15) | 1.12 (1.07–1.17) | 1.10 (1.05–1.15) |
| severe | 168,098 | 1,113 | 6.62 | 1.71 (1.53–1.92) | 1.68 (1.50–1.87) | 1.56 (1.40–1.75) | 1.52 (1.36–1.70) | 1.51 (1.35–1.69) | 1.63 (1.45–1.82) | 1.55 (1.39–1.74) |
| **spine** |  |  |  |  |  |  |  |  |  |  |
| mild | 2,000,332 | 1,601 | 0.80 | 1.06 (0.97–1.15) | 1.03 (0.95–1.12) | 1.00 (0.92–1.08) | 1.00 (0.92–1.08) | 1.00 (0.92–1.08) | 1.03 (0.95–1.12) | 0.99 (0.91–1.08) |
| moderate | 1,140,376 | 1,349 | 1.18 | 1.18 (1.08–1.30) | 1.14 (1.04–1.25) | 1.10 (1.00–1.20) | 1.11 (1.01–1.22) | 1.11 (1.01–1.22) | 1.14 (1.04–1.25) | 1.09 (1.00–1.20) |
| severe | 168,238 | 377 | 2.24 | 2.45 (2.02–2.96) | 2.31 (1.91–2.81) | 1.95 (1.60–2.37) | 1.83 (1.50–2.23) | 1.71 (1.40–2.09) | 2.13 (1.75–2.59) | 1.94 (1.59–2.36) |
| **wrist** |  |  |  |  |  |  |  |  |  |  |
| mild | 1,995,547 | 4,295 | 2.15 | 1.07 (1.02–1.12) | 1.05 (1.00–1.10) | 1.04 (0.99–1.09) | 1.04 (0.99–1.09) | 1.04 (0.99–1.09) | 1.05 (1.00–1.10) | 1.04 (0.99–1.09) |
| moderate | 1,137,548 | 2,920 | 2.57 | 1.13 (1.06–1.20) | 1.11 (1.04–1.18) | 1.10 (1.03–1.17) | 1.10 (1.04–1.17) | 1.10 (1.03–1.16) | 1.11 (1.04–1.18) | 1.10 (1.03–1.17) |
| severe | 167,791 | 522 | 3.11 | 1.35 (1.17–1.55) | 1.29 (1.12–1.49) | 1.25 (1.08–1.44) | 1.24 (1.07–1.43) | 1.22 (1.05–1.40) | 1.28 (1.10–1.47) | 1.25 (1.08–1.44) |
| **any** **site** |  |  |  |  |  |  |  |  |  |  |
| mild | 1,888,348 | 25,932 | 13.73 | 1.10 (1.07–1.12) | 1.07 (1.05–1.09) | 1.06 (1.04–1.08) | 1.06 (1.04–1.08) | 1.06 (1.04–1.08) | 1.07 (1.05–1.09) | 1.06 (1.04–1.08) |
| moderate | 1,061,083 | 16,293 | 15.36 | 1.18 (1.15–1.21) | 1.15 (1.12–1.18) | 1.13 (1.10–1.16) | 1.13 (1.10–1.16) | 1.13 (1.10–1.16) | 1.15 (1.12–1.18) | 1.13 (1.10–1.16) |
| severe | 152,771 | 2,734 | 17.90 | 1.37 (1.29–1.47) | 1.33 (1.25–1.42) | 1.24 (1.16–1.33) | 1.21 (1.13–1.29) | 1.19 (1.11–1.27) | 1.29 (1.21–1.38) | 1.24 (1.16–1.32) |

Total person years and number of fractures and crude rates per 1000 person years are shown.
^1^implicitly adjusted for age, sex, general practice, and date of cohort entry and additionally adjusted for time period (1997-2001, 2002-2006, 2007-2011, 2012-2016), quintiles of index of multiple deprivation and asthma.

Table E3: Incidence rates for different types of fractures for people with atopic eczema, confounder and cumulative steroid dose adjusted hazard ratios for fracture comparing those with atopic eczema to those without, and absolute rate differences (compared to people without atopic eczema).

| **Fracture** | **Incidence rate (per 1,000 p-yrs) in participants with atopic eczema** | **Hazard ratio^1^ comparing risk of fracture in people with atopic eczema to people without (99% confidence interval)^2^** | **Rate difference (per 1,000 p-yrs) (99% confidence interval) ^3^** |
| --- | --- | --- | --- |
| pelvis | 0.95 | 1.08 (1.01–1.15) | 0.07 (0.01–0.12) |
| hip | 4.14 | 1.09 (1.06–1.12) | 0.35 (0.23–0.46) |
| spine | 1.01 | 1.09 (1.03–1.16) | 0.08 (0.02–0.13) |
| wrist | 2.34 | 1.07 (1.03–1.11) | 0.16 (0.07–0.23) |
| any fracture | 14.49 | 1.10 (1.08–1.12) | 1.31 (1.11–1.52) |

Abbreviation: p-yrs=person-years
^1^for the risk of fracture comparing those with atopic eczema to those without atopic eczema.
^2^from models implicitly adjusted for age sex, general practice and date of cohort and additionally adjusted for time period (1997-2001, 2002-2006, 2007-2011, 2012-2016), quintiles of index of multiple deprivation, asthma and cumulative oral corticosteroid dose.
^3^The incidence rate in participants without atopic eczema used to calculate the rate difference is estimated as the incidence rate of those with atopic eczema multiplied by the inverse of the hazard ratio of the confounder adjusted model with cumulative dose (r*(1/HR)).

Text E1: Justifications for the inclusion of covariates, and the basis of their categorisation

**Deprivation**

Deprivation (conceptually related to lower socio-economic status) has been found to be associated with higher risk of low bone density and a lower risk of atopic eczema.^1–5^ We have therefore assumed that deprivation is a confounder, affecting multiple other variables. We used quintiles on the index of multiple deprivation (IMD), which aims to measure the relative deprivation between small geographic areas in England. The IMD is based on income, employment, education, health, crime, barriers to health and services, and living environment. ^6^ We used individual level IMD data, with practice-level IMD data used only when individual level data was not available.

**Calendar time**

We addressed changes over time in treatment, diagnostic and coding practices, and environmental exposures by considering calendar time as a confounder. We split calendar time into the following time bands: 1997-2001, 2002-2006, 2007-2011, 2012-2016. We included a deliberate split at 2012 as indicators of secondary fragility fracture prevention (related to osteoporosis) were added to the Quality and Outcomes Framework (QOF) that year.^7^ Inclusion of indicators in the QOF (which is responsible for incentivising the recording of specific codes in UK primary care ^8^) could have led to changes in recording and screening practices. ^9^

**Asthma**

Asthma is a common comorbidity in people with atopic eczema, being related to atopic eczema through a common ancestor (atopy or genetics).^10,11^ Inhaled corticosteroids are often used to treat asthma, which might lead to increased fracture risk and lower bone density at high doses,^12,13^ however there is no clear evidence of this effect at lower doses, even when used long term.^13–17^ Asthma serves as a proxy for inhaled corticosteroid use, as records of prescriptions only poorly reflect the actual use of inhaled corticosteroids, as these are often prescribed “to be taken as needed”. As with atopic eczema, due to the adverse effects linked to oral steroids, oral (or other systemic) steroids for the treatment of asthma should be reserved for scenarios where other treatments, including inhaled steroids, fail. Nevertheless, oral steroids were used extensively in the past for the treatment of asthma, especially before the advent of inhaled steroids, and are still used in many cases today.^18,19^ Therefore, we considered asthma to be a confounder, with oral steroid use likely accounting for most of its confounding effect, and inhaled steroid use and the effect of asthma on BMI^20^ playing smaller roles. Adjusting for asthma blocks these confounding paths (however a mediating path via oral steroids prescribed for atopic eczema remains open). We included asthma as a time updated covariate. Asthma is an indicator in the QOF, and should therefore be well captured in CPRD.^7^

**Body mass index**

Body mass index (BMI) is associated with osteoporosis, with underweight individuals at a higher risk. The protective effect of increasing weight is likely due to the strengthening effect on bones over time due to increased impact forces.^21,22^

BMI is likely to mediate some of the effect of atopic eczema on fractures. There are multiple plausible mechanisms through which BMI may mediate the effect of atopic eczema on fractures, including: diet, oral corticosteroids and physical activity.^21,23–25^ We therefore considered BMI as a mediator in this study and we included it as a covariate in models estimating the direct effect of atopic eczema.

Participants were categorised according to WHO guidelines into group of underweight (BMI<18.5 kg/m2), normal weight (18.5-25 kg/m2), overweight (25-30 kg/m2), and obese (>30 kg/m2). BMI was defined based on the record closest to index date with records within the year before to 1 month after index date preferred, then records from 1 month to 1 year after index date, then the most recent record prior to 12 months before index date, and then records within a year from index date.

**Smoking**

Smoking is associated with reduced bone density and therefore increased fracture risk.^26^ Individuals with atopic eczema are also more likely to be smokers.^27^ We therefore consider smoking is to be a mediator in our analyses. Participants were categorised as either never-smokers or ever-smokers (current or former smokers), based on the closest record of smoking status to cohort entry. Smoking status was defined based on the record closest to index date with records within the year before to 1 month after index date preferred, then records from 1 month to 1 year after index date, then the most recent record prior to 12 months before index date, and then records within a year from index date.

**Harmful alcohol use**

Harmful alcohol use is associated with fracture risk ^28–30^ and people with atopic eczema have also been found to be at higher risk of harmful alcohol use,^31^ making it a potential mediator. We used primary care records for relevant morbidity and prescription codes (for drugs used to deter people from alcohol use) that suggested harmful alcohol use and categorised individuals as harmful alcohol users from their first primary care record suggesting harmful alcohol use. We considered that harmful alcohol use was likely to mediate the effect of atopic eczema on fracture through increased rates of accidents and not via osteoporosis.

### Sensitivity analyses

Table E4: Sensitivity analysis using a different definition of cumulative dose.

|  |  | **HR (99% CI) for fracture risk comparing those with atopic eczema to those without, adjusted for confounders* and additionally adjusted for cumulative oral steroid dose** | | | | |
| --- | --- | --- | --- | --- | --- | --- |
| **Analysis** | **Justification** | **Pelvis** | **Hip** | **Spine** | **Wrist** | **Any site** |
| Main analysis | Included for comparison with results of sensitivity analysis. | 1.08 (1.01–1.15) | 1.09 (1.06–1.12) | 1.09 (1.03–1.16) | 1.07 (1.03–1.11) | 1.09 (1.08–1.11) |
| Analysis where **cumulative dose** for oral corticosteroid exposure was updated at the end of each respective prescription instead of at the beginning. | To explore if changing the definition has any major impact on the effect estimates. | 1.07 (1.01–1.14) | 1.09 (1.06–1.12) | 1.09 (1.03–1.16) | 1.07 (1.03–1.11) | 1.09 (1.08–1.10) |

*implicitly adjusted for age, sex, general practice, and date of cohort entry and additionally adjusted for time period (1997-2001, 2002-2006, 2007-2011, 2012-2016), quintiles of index of multiple deprivation and asthma.

Text E2: Sensitivity analysis: Inclusion of ethnicity as a covariate

We identified ethnicity (White, South Asian, Black, and other or mixed) from primary care (CPRD) and hospital admissions (HES) data using a previously developed algorithm.^32^ To explore if including ethnicity in models had an impact on the effect estimates we performed sensitivity analyses including ethnicity and restricting to individuals entering the cohort from 2006 (when ethnicity data was more likely to be complete, due to its recording being incentivised in the Quality and Outcomes Framework).^32^ Effect estimates from confounder adjusted and additionally corticosteroid adjusted models for the association between atopic eczema and pelvis, hip, spine and wrist fractures were attenuated and confidence intervals crossed the null when ethnicity was added to the models **(Table E5)**. However, effect estimates from confounder adjusted and additionally corticosteroids adjusted models using the restricted population also showed attenuated effect estimates without the addition of ethnicity as a covariate. Therefore, the results of the sensitivity analysis could be explained through differences in population structure rather than through an effect of ethnicity. We found that in the population with follow up from 2006 onwards, individuals were on average younger at baseline, compared to the main analysis **(Table E6)**.

Table E5: Sensitivity analysis additionally adjusting for ethnicity.

|  |  | **HR (99% CI) for fracture risk comparing those with atopic eczema to those without** | | | | | | | | | | |
| --- | --- | --- | --- | --- | --- | --- | --- | --- | --- | --- | --- | --- |
|  |  | **adjusted for confounders*** | | | | | | **adjusted for confounders* and additionally adjusted for cumulative oral steroid dose** | | | | |
| **Analysis** | **Justification** | **Pelvis** | **Hip** | **Spine** | **Wrist** | **Any site** | **Pelvis** | | **Hip** | **Spine** | **Wrist** | **Any site** |
| Main analysis | Included for comparison with results of sensitivity analysis. | 1.12 (1.05–1.19) | 1.11 (1.08–1.15) | 1.15 (1.08–1.22) | 1.08 (1.04–1.12) | 1.11 (1.09–1.13) | 1.08 (1.01–1.15) | | 1.09 (1.06–1.12) | 1.09 (1.03–1.16) | 1.07 (1.03–1.11) | 1.09 (1.08–1.11) |
| Main analysis restricting participants to those recruited from 2006 onwards. | Included for comparison with results of sensitivity analysis. | 0.96  (0.84–1.10) | 1.06  (1.00–1.13) | 1.05  (0.94–1.18) | 1.06  (0.98–1.14) | 1.10  (1.07–1.12) | 0.94  (0.82–1.08) | | 1.04  (0.98–1.11) | 1.00  (0.89–1.12) | 1.04  (0.97–1.12) | 1.08  (1.05–1.10) |
| Analysis additionally adjusting for **ethnicity** and restricting participants to those recruited from 2006 onwards. | To explore if including ethnicity in models has an impact on the effect estimates. Only included as a sensitivity analysis as data on ethnicity was frequently missing. Renumeration for the recording of ethnicity was introduced 2006 in the Quality and Outcomes framework, leading to more complete data on ethnicity from 2006 onwards.^32^ | 0.90  (0.79–1.03) | 1.03  (0.97–1.10) | 1.02  (0.91–1.15) | 1.03  (0.95–1.11) | 1.07  (1.05–1.10) | 0.89  (0.77–1.02) | | 1.01  (0.95–1.08) | 0.98  (0.87–1.10) | 1.01  (0.94–1.10) | 1.06  (1.03–1.08) |

*: implicitly adjusted for age, sex, general practice, and date of cohort entry and additionally adjusted for time period (1997-2001, 2002-2006, 2007-2011, 2012-2016), quintiles of index of multiple deprivation and asthma.

Table E6: Characteristics of participants from the study population used for the main analysis and from the study population restricted to those recruited from 2006 onwards, which was used for the sensitivity analysis additionally adjusting for ethnicity.

|  | **Main analysis sample** N=3,095,838 | | **Restricted to those recruited  from 2006 onwards** N=1,634,818 | |
| --- | --- | --- | --- | --- |
|  | **without atopic eczema** n=2,569,030 | **without atopic eczema** n=526,808 | **without atopic eczema** n=1,353,624 | **without atopic eczema** n=281,194 |
| **Follow up time,** p-yrs | 14,932,553 | 3,309,366 | 4,568,393 | 1,012,408 |
| **Median follow up time,** years (IQR) | 4.41 (2.00–9.64) | 5.02 (2.00–9.64) | 2.75 (1.14–5.16) | 3.03 (1.28–5.54) |
| **Sex** female, n (%) | 1,489,261 (58.0%) | 308,071 (58.5%) | 762,689 (56.3%) | 160,753 (57.2%) |
| **Age** (years), n (%) |  |  |  |  |
| 18–39 | 1,217,722 (47.4%) | 246,596 (46.8%) | 715,467 (52.9%) | 145,762 (51.8%) |
| 40–49 | 351,927 (13.7%) | 69,696 (13.2%) | 180,172 (13.3%) | 35,480 (12.6%) |
| 50–59 | 329,007 (12.8%) | 63,943 (12.1%) | 158,152 (11.7%) | 30,897 (11.0%) |
| 60–69 | 303,790 (11.8%) | 61,902 (11.8%) | 147,092 (10.9%) | 30,709 (10.9%) |
| 70+ | 366,584 (14.3%) | 84,671 (16.1%) | 152,741 (11.3%) | 38,346 (13.6%) |
| **BMI** (kg/m^2^), n (%) |  |  |  |  |
| normal (18.5-25) | 828,367 (32.2%) | 172,446 (32.7%) | 413,000 (30.5%) | 84,772 (30.1%) |
| underweight (<18.5) | 185,784 (7.2%) | 37,756 (7.2%) | 101,069 (7.5%) | 20,498 (7.3%) |
| overweight (25-30) | 667,277 (26.0%) | 143,919 (27.3%) | 338,930 (25.0%) | 73,334 (26.1%) |
| obese (>30kg) | 393,529 (15.3%) | 92,507 (17.6%) | 224,251 (16.6%) | 53,622 (19.1%) |
| missing | 494,073 (19.2%) | 80,180 (15.2%) | 276,374 (20.4%) | 48,968 (17.4%) |
| **Smoking**, n (%) |  |  |  |  |
| never | 1,293,983 (50.4%) | 266,134 (50.5%) | 662,868 (49.0%) | 136,691 (48.6%) |
| ever | 1,125,627 (43.8%) | 246,782 (46.8%) | 633,388 (46.8%) | 139,152 (49.5%) |
| missing | 149,420 (5.8%) | 13,892 (2.6%) | 57,368 (4.2%) | 5,351 (1.9%) |
| **IMD^1^**, n (%) |  |  |  |  |
| 1(least deprived) | 611,904 (23.8%) | 126,806 (24.1%) | 311,069 (23.0%) | 66,174 (23.5%) |
| 2 | 589,313 (22.9%) | 120,946 (23.0%) | 306,271 (22.6%) | 64,133 (22.8%) |
| 3 | 508,469 (19.8%) | 103,646 (19.7%) | 266,234 (19.7%) | 55,080 (19.6%) |
| 4 | 489,144 (19.0%) | 100,430 (19.1%) | 267,687 (19.8%) | 54,947 (19.5%) |
| 5 (most deprived) | 370,200 (14.4%) | 74,980 (14.2%) | 202,363 (14.9%) | 40,860 (14.5%) |

Abbreviation: IQR: interquartile range; p-yrs: person-years; ^1^Quintiles of the Index of multiple deprivation (IMD)

### References

1. Golding J, Peters TJ. The epidemiology of childhood eczema: I. A population based study of associations. Paediatric and Perinatal Epidemiology. 1987;1:67–79.

2. Peters TJ, Golding J. The epidemiology of childhood eczema: II. Statistical analyses to identify independent early predictors. Paediatric and Perinatal Epidemiology. 1987;1:80–94.

3. Barbee RA. Immediate Skin-Test Reactivity in a General Population Sample. Annals of Internal Medicine. 1976;84:129.

4. Crandall CJ, Merkin SS, Seeman TE, Greendale GA, Binkley N, Karlamangla AS. Socioeconomic status over the life-course and adult bone mineral density: The Midlife in the U.S. Study. Bone. 2012;51:107–13.

5. Hammer-Helmich L, Linneberg A, Thomsen SF, Glümer C. Association between parental socioeconomic position and prevalence of asthma, atopic eczema and hay fever in children. Scandinavian Journal of Public Health. 2014;42:120–7.

6. Jordan H. The Index of Multiple Deprivation 2000 and accessibility effects on health. Journal of Epidemiology & Community Health. 2004;58:250–7.

7. Primary Care Strategy and NHS Contracts Group. 2019/20 General Medical Services (GMS) contract Quality and Outcomes Framework (QOF). 2019.

8. Forbes LJ, Marchand C, Doran T, Peckham S. The role of the Quality and Outcomes Framework in the care of long-term conditions: a systematic review. British Journal of General Practice. 2017;67:e775–84.

9. NICE Clinical guidance. Osteoporosis: assessing the risk of fragility fracture [Internet]. 2017. Available from: www.nice.org.uk/guidance/cg146

10. Lowe KE, Mansfield KE, Delmestri A, Smeeth L, Roberts A, Abuabara K, et al. Atopic eczema and fracture risk in adults: A population-based cohort study. Journal of Allergy and Clinical Immunology. 2020;145:563-571.e8.

11. Silverberg JI, Hanifin JM. Adult eczema prevalence and associations with asthma and other health and demographic factors: A US population–based study. Journal of Allergy and Clinical Immunology. 2013;132:1132–8.

12. Van Staa TP, Leufkens HGM, Cooper C. Use of Inhaled Corticosteroids and Risk of Fractures. Journal of Bone and Mineral Research. 2001;16:581–8.

13. Hubbard RB, Smith CJP, Smeeth L, Harrison TW, Tattersfield AE. Inhaled Corticosteroids and Hip Fracture: A Population-based Case–Control Study. American Journal of Respiratory and Critical Care Medicine. 2002;166:1563–6.

14. Wong CA, Walsh LJ, Smith CJ, Wisniewski AF, Lewis SA, Hubbard R, et al. Inhaled corticosteroid use and bone-mineral density in patients with asthma. The Lancet. 2000;355:1399–403.

15. Langhammer A, Norjavaara E, de Verdier MG, Johnsen R, Bjermer L. Use of inhaled corticosteroids and bone mineral density in a population based study: the Nord-Trøndelag Health Study(the HUNT Study). Pharmacoepidemiology and Drug Safety. 2004;13:569–79.

16. Chee C, Sellahewa L, Pappachan JM. Inhaled Corticosteroids and Bone Health. The Open Respiratory Medicine Journal. 2015;8:85–92.

17. Loke YK, Gilbert D, Thavarajah M, Blanco P, Wilson AM. Bone mineral density and fracture risk with long-term use of inhaled corticosteroids in patients with asthma: systematic review and meta-analysis. BMJ Open. 2015;5:e008554.

18. Gaga M, Zervas E. Oral steroids in asthma: a double-edged sword. European Respiratory Journal. 2019;54:1902034.

19. Ramsahai JM, Wark PA. Appropriate use of oral corticosteroids for severe asthma. Medical Journal of Australia [Internet]. 2018 [cited 2020 Jul 26];209. Available from: https://onlinelibrary.wiley.com/doi/abs/10.5694/mja18.00134

20. Peters U, Dixon A, Forno E. Obesity and Asthma. The Journal of allergy and clinical immunology. 2018;141:1169.

21. Iwaniec UT, Turner RT. Influence of body weight on bone mass, architecture and turnover. Journal of Endocrinology. 2016;230:R115–30.

22. Felson DT, Zhang Y, Hannan MT, Anderson JJ. Effects of weight and body mass index on bone mineral density in men and women: The framingham study. Journal of Bone and Mineral Research. 2009;8:567–73.

23. Christie L, Hine RJ, Parker JG, Burks W. Food Allergies in Children Affect Nutrient Intake and Growth. Journal of the American Dietetic Association. 2002;102:1648–51.

24. Kim A, Silverberg JI. A systematic review of vigorous physical activity in eczema. British Journal of Dermatology. 2016;174:660–2.

25. Chilibeck PD, Sale DG, Webber CE. Exercise and Bone Mineral Density: Sports Medicine. 1995;19:103–22.

26. Ward KD, Klesges RC. A meta-analysis of the effects of cigarette smoking on bone mineral density. Calcified Tissue International. 2001;68:259–70.

27. Kantor R, Kim A, Thyssen JP, Silverberg JI. Association of atopic dermatitis with smoking: A systematic review and meta-analysis. Journal of the American Academy of Dermatology. 2016;75:1119-1125.e1.

28. Berg KM, Kunins HV, Jackson JL, Nahvi S, Chaudhry A, Harris KA, et al. Association Between Alcohol Consumption and Both Osteoporotic Fracture and Bone Density. The American Journal of Medicine. 2008;121:406–18.

29. Hoidrup S, Gronbaek M, Gottschau A, Lauritzen JB, Schroll M, Copenhagen Centre for Prospective Population Studies. Alcohol Intake, Beverage Preference, and Risk of Hip Fracture in Men and Women. American Journal of Epidemiology. 1999;149:993–1001.

30. Kanis JA, Johansson H, Johnell O, Oden A, De Laet C, Eisman JA, et al. Alcohol intake as a risk factor for fracture. Osteoporosis International. 2005;16:737–42.

31. Al‐Jefri K, Newbury‐Birch D, Muirhead CR, Gilvarry E, Araújo‐Soares V, Reynolds NJ, et al. High prevalence of alcohol use disorders in patients with inflammatory skin diseases. British Journal of Dermatology. 2017;177:837–44.

32. Mathur R, Bhaskaran K, Chaturvedi N, Leon DA, vanStaa T, Grundy E, et al. Completeness and usability of ethnicity data in UK-based primary care and hospital databases. Journal of Public Health. 2014;36:684–92.
